# Seroprevalence and risk factors of porcine cysticercosis in Mpwapwa district, eastern-Central Tanzania

**DOI:** 10.1016/j.parepi.2025.e00445

**Published:** 2025-06-30

**Authors:** Justine Daudi Maganira, Noel Mark Makwinya, Beda John Mwang'onde

**Affiliations:** a Department of Biosciences, Sokoine University of Agriculture, P.O. Box 3038, Morogoro, Tanzania; b Department of Curriculum and Instruction, School of Education, Sokoine University of Agriculture, P.O. Box 3038, Morogoro, Tanzania

**Keywords:** *Taenia solium*, Seroprevalence, Porcine cysticercosis, Antigen, Mpwapwa, Tanzania

## Abstract

Porcine cysticercosis, caused by the larval stage of *Taenia* species, poses significant health and economic challenges in low and lower-middle-income regions. This study assessed the seroprevalence and risk factors associated with the transmission of porcine cysticercosis in Mpwapwa District, eastern-central Tanzania, using a commercial antigen enzyme linked immunosorbent assay (Ag- ELISA). A cross-sectional survey was conducted in Gulwe, Ving'hawe, and Igovu villages during the dry season in June 2024. Blood samples were collected from 159 household pigs, and structured questionnaires were administered to heads or representatives of 51 pig-keeping households to identify potential risk factors. Serum samples from 29 pigs tested positive for porcine cysticercosis in the Ag-ELISA assay with an apparent prevalence of 18.24 % (95 % CI: 12.57 %–25.13 %), and estimated true prevalence of 17.91 % (95 % CI: 12.66 %–24.67 %). The apparent prevalence of porcine cysticercosis varied across the villages with Gulwe showing the highest prevalence (22.58 %; 95 % CI: 12.93 %–34.79 %), followed by Ving'hawe (21.21 % 95 % CI: 8.98 %–38.91 %), and Igovu (12.50 %; 95 % CI: 5.55 %–23.15 %). Female pigs exhibited a relatively higher seropositivity rate (13.84 %; 95 % CI: 8.88 %–20.20 %) compared to male pigs (4.40 %; 95 % CI: 1.79 %–8.86 %) although this difference was not statistically significant (χ^2^ = 0.629, *p*-value = 0.428). DNA extracted from cysticerci isolated from infected pigs slaughter in the study villages was confirmed via gel electrophoresis to belong to *Taenia solium*. Seropositivity was higher in adult pigs (14.47 %; 95 % CI: 9.84 %–20.77 %) than in growers (3.77 %; 95 % CI: 1.74 %–7.99 %), but this difference was not statistically significant (χ^2^ = 0.385, *p* = value = 0.535). Risk factor analysis identified the pig management system (OR = 2.47, *p* = 0.005), household pig herd size (OR = 3.08, *p* = 0.003), pig pen design (OR = 2.49, *p* = 0.002), feed source (OR = 3.08, *p* = 0.000), ignorance of porcine cysticercosis (OR = 1.57, *p* = 0.031) and presence of open-field defecation (OR = 1.47, *p* = 0.025) as significant contributors to the transmission of porcine cysticercosis. This study identifies a significant burden of porcine cysticercosis in Mpwapwa District, highlighting the need for effective control strategies to combat this zoonotic disease, protect smallholder livelihoods, and align with the WHO's 2030 targets for intensified *T. solium* control.

## 1 Introduction

Porcine cysticercosis, caused by the larval stage (cysticerci) of *Taenia solium*, is a major veterinary and public health concern. This parasitic infection affects pig health and productivity, compromising food safety, food security, and human health (Nkwengulila, 2014; Trevisan et al., 2018). Humans serve as definitive hosts for *T. solium*, harbouring adult tapeworms in their intestines and shedding eggs that infect pigs, thereby continuing the transmission cycle (Flisser, 2013; Okamoto and Ito, 2013). In contrast, *T. hydatigena* primarily uses dogs and other canids as definitive hosts, with pigs and other livestock species serving as intermediate hosts (Braae et al., 2015).

Cysticercosis poses a significant threat to both public health and livelihoods. According to WHO (2022), *T. solium* is responsible for 30 % of epilepsy cases in endemic areas where humans and free-roaming pigs coexist, with this proportion reaching 70 % in high-risk communities. Over 80 % of the 50 million people affected by epilepsy globally live in low- and lower-middle-income countries. In 2015, the WHO Foodborne Disease Burden Epidemiology Reference Group identified *T. solium* as a leading cause of mortality from foodborne diseases, accounting for an estimated 2.8 million disability-adjusted life-years (DALYs). Epilepsy further exacerbates the burden by contributing to stigma, reduced quality of life, and, in severe cases, death (Coral-Almeida et al., 2014; Mwape et al., 2015). In pigs, porcine cysticercosis results in carcass condemnation or reduced market value, causing significant economic losses for smallholder farmers (Praet et al., 2009). These challenges are particularly severe in rural sub-Saharan Africa, where pig farming is among the key sources of income and protein.

The prevalence of porcine cysticercosis varies widely, ranging from less than 1 % to over 50 %, depending on local farming practices, sanitation, and access to veterinary and public health services (Assana et al., 2013; Ngowi et al., 2019). Transmission is closely linked to traditional pig management systems, where pigs roam freely and scavenge for food, increasing their exposure to *T. solium* eggs in contaminated environments. Insufficient meat inspection and limited public health awareness exacerbate the problem (Phiri et al., 2003).

Despite reports of porcine cysticercosis in several Tanzanian districts (Maganira et al., 2018; Maganira et al., 2019; Ngowi et al., 2019), many high-density pig-farming regions remain unsurveyed. Mpwapwa District, located in eastern-central Tanzania, is one such area where the epidemiology of the disease and its associated risk factors remain poorly understood. Targeted research in this region is crucial to inform effective control and prevention strategies, safeguarding public health, improving animal welfare, and protecting smallholder livelihoods.

## 2 Materials and methods

### 2.1 Study area

This study was conducted in three villages: Gulwe, Ving'hawe, and Igovu, located in Mpwapwa District, Dodoma, eastern-central Tanzania. This region, characterized by high pig populations and traditional management practices among smallholder farmers, represents a critical area for such research on porcine cysticercosis. Mpwapwa District lies between latitude 6°00“ and 7°30”S and longitude 35°45“ and 37°00”E, approximately 120 km from Dodoma City. The District covers an area of 7379 km^2^.

Mpwapwa is bordered to the north by Kongwa District, to the east by Morogoro region, to the south by Iringa region, and to the west by Chamwino District. It experiences a dry savannah climate with an annual average temperature of 27 °C and rainfall ranging from 600 mm to 1200 mm per year. Compared to other Districts in the Dodoma region, Mpwapwa receives relatively higher rainfall annually.

The District is divided into three administrative divisions: Mpwapwa, Kibawe, and Rudi, comprising 33 wards and 113 villages. The human population in Mpwapwa is estimated at 403,247 (URT, 2022). Key economic and livelihood activities in Mpwapwa include food crop farming, livestock keeping, fishing, and mining, with smallholder pig farming on the rise.

### 2.2 Study design and sample size estimation

A cross-sectional survey was conducted during the dry season in June 2024. Blood serum samples were collected from live pigs raised by smallholder farmers and tested for circulating taeniid antigens, which are indicative of porcine cysticercosis. Risk factor data were gathered through structured questionnaires administered to heads or representatives of pig-keeping households.

The sample size for blood serum testing was calculated using the formula *n* = *Z*^2^*PQ*/*L*^2^ (Thrusfield, 2005), where *n* represents the number of pigs included in the study, *Z* corresponds the *Z*-score for a 95 % confidence interval, *P* is the expected prevalence of porcine cysticercosis in Mpwapwa District, determined to be 12 % based on a study conducted in a neighbouring District (Maganira et al., 2019), *Q* is the proportion of pigs estimated to be free from porcine cysticercosis (that is *Q* = 1 – *P*), and *L* is the desired precision, set at 5 %. Based on these parameters, the estimated sample size from the three study villages was 162 pigs (54 pigs from each village). The calculation did not account for household clustering, and therefore, no adjustments were made to the sample size.

Households were eligible for inclusion only if they kept at least two pigs at the time of this study, and the questionnaire sample size was equivalent to the number of households from which blood serum samples were collected. Additionally, as part of the original study plan, cysticerci were to be collected from pigs found to be naturally infected at slaughter within the study area during the data collection period for molecular confirmation.

### 2.3 Study household selection

The study targeted six wards with the highest pig populations, as identified through preliminary information obtained from a District veterinary officer. A simple random sampling method was used to select three of the six wards. Small pieces of paper with the names of the six wards were prepared; and through blind-folded picking, three wards: Godegode, Ving'hawe, and Massa; were randomly selected. Subsequently, one village was randomly selected from each chosen ward following the same process. The selected villages included Gulwe (Godegode ward), Ving'aHwe (Ving'aHwe ward), and Igovu (Massa ward). Within each village, households were eligible for inclusion if they kept at least two pigs at the time of data collection and if the household head or a representative provided informed consent to participate in the study. The number of households selected ranged from 8 to 28 per village. In total, 51 household heads or their representatives from the three villages participated in the study.

### 2.4 Data collection

Blood samples were collected from the auricular vein of live household pigs aged at least 4 months. However, pigs younger than 3 months, pregnant sows, and nursing sows with litters under 2 months old were excluded. Pigs were categorized based on age as follows: piglets (2 months and below), weaners (3 months), Growers (between 4 and 5 months) and adults (between 6 months and above). In households with more than four pigs, random sampling was employed to select only four pigs.

To facilitate blood collection, pigs were restrained using a hog catcher. The auricular vein was cleaned with 70 % ethanol before sampling, and approximately 4 ml of blood was drawn using a 23-gauge hypodermic needle. Bleeding at the sampling site was stopped using sterile gauze. Blood samples were placed in 5 ml plain vacutainer tubes, labeled, and stored in a cooler box at 4 °C. Serum was extracted within 4 to 8 h of collection by centrifugation at 2000 ×*g* for 10 min. Clear serum samples were stored in cryogenic vials at −20 °C until further analysis.

In addition to blood sampling, suspected viable *Taenia* species cysticerci were obtained from three pigs during slaughter. These samples were collected during meat inspections conducted on the second and fourth days of data collection in June 2024. Cysticerci were recovered from the brain, diaphragm, and heart, and separately placed in 50 ml Falcon tubes containing 70 % ethanol for molecular identification using conventional polymerase chain rection (PCR).

To assess risk factors for porcine cysticercosis transmission, a structured questionnaire was administered to the head or representative of each of the 51 surveyed households. The questionnaire design was informed by previous studies conducted in similar settings by Maganira et al. (2019) and Sanga et al. (2023), with modifications tailored to the specific context and objectives of the current study. An expert who had participated in those studies reviewed and guided the adaptation process, thereby enhancing the construct-related, content-related, and face-related validity of the tool. Although the questionnaire was not formally pretested, expert input helped refine the questions to ensure they were understandable, relevant, and capable of yielding reliable data. The questionnaire focused on key risk factors, including pig husbandry practices, access to sanitary facilities, and knowledge of the disease, to support comprehensive data collection on porcine cysticercosis transmission in the study area.

### 2.5 ELISA antigen detection

Serum samples were analysed for porcine cysticercosis at the College of Veterinary Medicine and Biomedical Sciences, Sokoine University of Agriculture, Morogoro, Tanzania, using a commercially available genus-specific antigen enzyme linked immunosorbent assay (Ag-ELISA) as per the manufacturer's protocol (apDia, Belgium). The Ag-ELISA sensitivity and specificity are reported to be 100 % and 99.6 %, respectively (apDia, 2004). Samples were run in duplicates within a single analytical run to ensure consistency and minimize potential variability between runs. Positive and negative controls, along with test serum samples, were pretreated with trichloroacetic acid (TCA). Each pretreated sample (100 μl) was added to the antibody-coated microtiter wells, sealed with a microplate sealer, and incubated at 37 °C for 15 min while shaking at 700 rpm. After incubation, the microtiter wells were washed five times with 300 μl washing solution to remove unbound antigens, and excess fluid was blotted off using tissue paper. Subsequently, 100 μl of conjugate solution was added to the wells, sealed, and incubated at 37 °C for 15 min with shaking at 700 rpm. Following another washing step, 100 μl of chromogen solution was added to each well, and the wells were sealed and incubated at room temperature for 15 min. The reaction was stopped by adding 50 μl of stopping solution, and optical density (OD) was measured at 450 nm using a microplate reader.

The cut-off value was calculated as the mean OD of negative controls (ODneg) multiplied by 3.5. The antigen index (Ag-index) of each sample was calculated as the OD value divided by the cut-off value. Results were classified as positive if the Ag-index was ≥1.3, negative if the Ag-index was ≤0.8, and doubtful if the Ag-index was between 0.8 and 1.3.

### 2.6 Cysticerci molecular analysis

Total DNA was extracted from suspected *Taenia* species cysticerci collected during slaughter using the QIAamp DNA Mini Kit (Qiagen, Hilden, Germany) in the Parasitology Section Laboratory, Department of Biosciences, College of Natural and Applied Sciences, Sokoine University of Agriculture, Morogoro, Tanzania. The extraction followed the manufacturer's protocol. Molecular characterization was performed using conventional PCR on a MyCycler thermal cycler (Bio-Rad Laboratories) with specific primers targeting the mitochondrial *cytochrome c oxidase subunit I* (cox1) gene, as described by Maganira et al. (2019), 2020). The forward primer (Tsol_R: 5′-ATTCTTCCGGGGTTTGGTAT-3′) and reverse primer (Tsol_R: 5′-CCTTAATCCCCGTAGGCA-3′), synthesized by Eurofins Genomics, were used for amplification of the extracted DNA. PCR reactions were prepared in a total volume of 25 μl, containing 3 μl of template DNA, 0.5 μM of each primer, 2.5 μl of 10× PCR buffer, 2 μl of MgCl2, 0.65 μl of dNTPs, 0.13 μl of AmpliTaq Gold DNA polymerase, and 15.7 μl of molecular-grade H2O.

The PCR cycling conditions were as follows: an initial denaturation at 95 °C for 10 min, followed by 40 cycles of denaturation at 95 °C for 30 s, annealing at 50 °C for 30 s, and extension at 72 °C for 1 min. The reaction concluded with a final elongation at 72 °C for 5 min. Positive and no-template controls (molecular-grade H_2_O) were included in each run. Amplified PCR products (5 μl) were analysed using 1 % agarose gel electrophoresis, which separates DNA fragments based on size, to visualise and identify species-specific genetic markers for *T. solium*.

### 2.7 Statistical data analysis

Statistical data analyses were performed using R version 4.3.3 (2024-02-29 ucrt), SPSS version 24, and Microsoft Office Excel 2019. Descriptive statistics were applied to determine the prevalence of porcine cysticercosis in the examined pigs. The Epi Tools package was utilised to calculate the estimated true prevalence of porcine cysticercosis with a 95 % confidence interval, incorporating the sensitivity (100 %) and specificity (99.6 %) of the Ag-ELISA, as reported by the manufacturer (apDia, 2004). Associations between risk factors and Ag-ELISA results were analysed using the Pearson Chi-square Test or Fisher's Exact Test, and the strengths of associations were quantified with odds ratios. Risk factors with *p*-values less than 0.05 were considered statistically significant.

### 2.8 Ethical approval

This study received ethical approval from the Sokoine University of Agriculture Research Ethics Committee (SUA-REC) and the Directorate of Postgraduate Studies, Research, Technology Transfer, and Consultancies (reference number DPRTC/R/126/SOET/3/2023). Research clearance was also granted by the Tanzania President's Office, Regional Administration, and Local Government (PO-RALG) ministry (reference number JC.156/254/01). Furthermore, informed consent was obtained from the head or a representative of each participating household prior to their involvement in the study.

## 3 Results

### 3.1 Study subjects

Initially, the study aimed to collect samples from 162 pigs, with an equal distribution of 54 pigs per village. However, the final sample size varied due to some households in Ving'hawe not providing consent. As a result, 64 pigs (40.25 %; 95 % CI: 32.56 %–48.31 %) were sampled from Igovu, 62 (38.99 %; 95 % CI: 31.37 %–47.04 %) from Gulwe, and 33 (20.75 %; 95 % CI: 14.74 %–27.89 %) from Ving'hawe. The observed differences in the number of pigs examined across the villages were statistically significant (χ^2^ = 11.358, *p* = 0.003). Most of the examined pigs were female (*n* = 108; 67.92 %; 95 % CI: 60.07 %–75.10 %), significantly higher than male pigs (*n* = 51; 32.08 %; 95 % CI: 24.90 %–37.93 %) (χ^2^ = 20.434, *p* < 0.001). Regarding age categories, most pigs were adults (*n* = 116; 72.96 %; 95 % CI: 65.35 %–79.69 %), followed by growers (*n* = 43; 27.04 %; 95 % CI: 20.31 %–34.65 %) (χ^2^ = 33.516, p < 0.001). The mean age of the examined pigs was 8.3 months, ranging from 4 to 44 months.

A total of 51 respondents participated in the survey, with 42 females (82.4 %; 95 % CI: 70.85 %–90.84 %) and 9 males (17.6 %; 95 % CI: 9.16 %–29.15 %). The difference in gender distribution was statistically significant (χ^2^ = 23.712, *p* < 0.001). Regarding age, 11 respondents (21.6 %; 95 % CI: 12.55 %–34.12 %) were classified as youth (18–25 years), while 40 respondents (78.4 %; 95 % CI: 65.88 %–87.45 %) were adults (26 years and above). The difference in age distribution was statistically significant (χ^2^ = 15.314, *p* < 0.001). In terms of education level, 35 respondents (68.6 %; 95 % CI: 54.94 %–79.94 %) had formal education, while 16 (31.4 %; 95 % CI: 20.06 %–45.06 %) had informal education. The observed difference in educational levels was statistically significant (χ^2^ = 9.941, *p* = 0.002). Among the respondents, 5 farmers (9.8 %; 95 % CI: 3.07 %–19.25 %) had less than 2 years of farming experience, while 46 farmers (90.2 %; 95 % CI: 80.75 %–96.93 %) had 3 or more years of farming experience, with the difference in farming experience distribution being statistically significant (χ^2^ = 31.235, *p* < 0.001).

### 3.2 Seropositivity for Taenia species antigens

Of the 159 serum samples from the surveyed pigs in Mpwapwa, 29 tested positive for porcine cysticercosis in the Ag-ELISA with an apparent prevalence (AP) of 18.24 % (95 % CI: 12.57 %–25.13 %), and estimated true prevalence (TP) of 17.91 % (95 % CI: 12.66 %–24.67 %). Seropositivity in pigs varied among villages with the highest prevalence recorded in pigs from Gulwe, followed by Ving'hawe and Igovu (Table 1). Porcine cysticercosis infection rate between villages was not statistically different (χ^2^ = 3.8693, *p*-value = 0.145).

**Table 1:** Prevalence of porcine cysticercosis from three villages in Mpwapwa District.

Village	House	Pigs	House+	Pigs+	AP (%)	95 % CI	ETP (%)	95 % CI
Gulwe	28	62	11	14	22.58	12.93–34.79	22.27	13.61–34.14
Igovu	8	64	4	8	12.50	5.55–23.15	12.15	6.10–22.46
Ving'hawe	15	33	7	7	21.21	8.98–38.91	20.90	10.32–37.56
Total	51	159	22	29	18.24	12.57–25.13	17.91	12.66–24.67

**Key:** House = Household; House+ = Household with positive pigs; Pigs+ = Positive pigs; AP = Apparent prevalence; ETP = Estimated true prevalence; % = percentage; CI = Confidence interval.

More female pigs (*n* = 22) tested positive for porcine cysticercosis in the Ag-ELISA with an AP of 13.84 % (95 % CI: 8.84 %–19.84 %) and an TP of 13.49 % (95 % CI: 8.95 %–19.74 %) compared to male pigs (*n* = 7) with an AP of 4.40 % (95 % CI: 1.78 %–8.84 %) and a TP of 4.02 % (95 % CI: 1.76 %–8.44 %), though this difference was not statistically significant (χ^2^ = 0.629, *p* = 0.428). Seropositivity was also higher in adult pigs (*n* = 23; AP: 14.47 %; 95 % CI: 9.84 %–20.77 %; and TP: 14.12 %; 95 % CI: 9.47 %–20.45 %) compared to growers (*n* = 6; AP: 3.77 %; 95 % CI: 1.74 %–7.99 %; and TP: 3.39 %; 95 % CI: 1.35 %–7.62 %). However, the differences in seropositivity among age groups were not statistically significant (χ^2^ = 0.385, *p* = 0.535).

### 3.3 Cysticerci molecular identity

As part of the original study plan, cysticerci were collected from pigs found to be naturally infected at slaughter within the study area during the data collection period. Unfortunately, only three pigs were found infected at slaughter during this period. DNA extracted from cysticerci isolated from the brain, diaphragm, and heart of these three infected pigs was confirmed by gel electrophoresis to belong to *T. solium.*

### 3.4 Risk factors for porcine cysticercosis seropositivity

The associations between risk factors and porcine cysticercosis seropositivity, evaluated using Pearson Chi-square or Fisher's Exact Test, are presented in Table 2. The strengths of these associations, expressed as odds ratios (OR) with 95 % confidence intervals (CI), are summarized in Table 3. Briefly, the identified risk factors significantly associated with the transmission of porcine cysticercosis in Mpwapwa District include pig herd size (with households having ≥6 pigs showing a higher risk), pig management system (with semi-intensive systems posing a greater risk), pigpen design (porous pens being more associated with the disease), pig feed source (local feed increasing the likelihood of infection), open defecation (increasing the risk when practiced), and ignorance of porcine cysticercosis (those unaware of the disease having a higher likelihood of infection).

**Table 2:** Risk factors associated with porcine cysticercosis transmission in Mpwapwa District, Tanzania.

Factor	Level	#Households	% + Households	*p*-value
Education	Informal	16	31.8	0.952
	Formal	35	68.2	
Farmer's Experience	≤ 2 years	5	4.5	0.271
	≥ 3 years	46	95.5	
Pig herd size	≤ 5 pigs	31	36.4	0.002
	≥ 6 pigs	20	63.6	
Pig management system	Semi-intensive	23	68.2	0.004
	Intensive	28	31.8	
Pigpen design	Porous	26	77.3	0.001
	Non-porous	25	22.7	
Pig feed source	Local feed	22	100	0.000
	Commercial feed	0	0	
Toilet type	Pit latrine	30	59.1	0.973
	Improved toilet	21	40.9	
Open defecation	Yes	38	90.9	0.025
	No	13	9.1	
PC ignorance	Yes	35	86.4	0.031
	No	16	13.6	

**Key:** #Households = Number of surveyed households; %Prev = Percentage prevalence of seropositive pigs; % + Households = Percentage of households with seropositive pigs; PC = Porcine cysticercosis.

**Table 3:** Association between risk factors of porcine cysticercosis transmission using Fisher's Exact Test *p*-values and Odds Ratios.

Predictor	Level	Odds Ratio	95 % CI	*p*-value
Education	Informal	1.025	0.45–2.32	1.000
	Formal	Ref.		
Farmer's experience	≤ 2 years	0.33	0.04–2.75	0.375
	≥ 3 years	Ref.		
Pig herd size	≥ 6 pigs	3.08	1.41–6.71	0.003
	≤ 5 pigs	Ref.		
Pig management system	Semi-intensive	2.47	1.28–4.76	0.005
	Intensive	Ref.		
Pigpen design	Porous	2.49	1.38–4.48	0.002
	Non-Porous	Ref.		
Pig feed source	Local feed	3.08	1.77–5.33	0.000
	Commercial feed	Ref.		
Open defecation	Yes	1.47	1.07–2.00	0.025
	No	Ref.		
Toilet type	Pit latrine	1.01	0.63–1.60	1.000
	Improved toilet	Ref.		
PC ignorance	Yes	1.57	1.08–2.26	0.031
	No	Ref.		

**Key:** OR = Odds Ratios; 95 % CI = 95 % confidence intervals; PC = porcine cysticercosis.

## 4 Discussion

This study investigated the prevalence of porcine cysticercosis and its key transmission risk factors in Mpwapwa District, Tanzania, eastern-central Tanzania. The estimated true prevalence, based on the apDia cysticercosis Ag-ELISA, was 17.91 %, highlighting the significance of porcine cysticercosis in the region. Molecular analysis of cysticerci from various carcass organs in three infected pigs confirmed their identity as *T. solium*. This molecular confirmation establishes that *T. solium* porcine cysticercosis is present in Mpwapwa villages. However, due to cross-reactivity and inability of the Ag-ELISA to differentiate *Taenia* species, the potential contribution of other taeniid worms, such as *T. hydatigena*, to these findings requires further investigation (Akoko et al., 2019; Dermauw et al., 2016).

The observed prevalence of porcine cysticercosis in this study was lower than that reported in Nyasa District in the southern highlands (Shonyela et al., 2017) and Babati District in the northern highlands of Tanzania (Kavishe et al., 2017). Similarly, higher prevalence rates have been documented in neighbouring countries including Kenya (Eshitera et al., 2012). Nevertheless, a lower prevalence rate than recorded here has also been reported (Maganira et al., 2018). However, the findings of this study align with those from nearby Kongwa District (Maganira et al., 2019). Additionally, prevalence varied across the three villages, with Gulwe having the highest rate, followed by Ving'hawe and Igovu. Despite these differences, statistical analysis revealed no significant variation, suggesting a relatively uniform risk of infection across villages. This lack of statistical significance could stem from limited sample sizes or factors, such as variations in pig management practices. Differences in porcine cysticercosis prevalence across districts and countries may be attributed to factors such as pig management systems, hygiene and sanitation standards, meat inspection practices, awareness of *Taenia* zoonoses, and socio-cultural behaviors. Differences in these factors may result into differences in exposure to *T. solium* or other *Taenia* species eggs. Variations in study design, and sample size, likely also contribute to the observed disparities.

Gender-based differences in prevalence were also investigated. The Ag-ELISA results showed a higher prevalence of porcine cysticercosis in female pigs compared to male pigs. However, this difference was not statistically significant, suggesting that gender is not a strong risk factor for infection in the study area. Similar findings have been reported in other studies (Akoko et al., 2019), where no significant gender-based differences in prevalence were observed. The higher prevalence in female pigs observed here may be attributed to the larger sample size of female pigs compared to males in this study.

Age-related differences in seropositivity were also observed, with adult pigs showing a higher prevalence compared to growers. Despite this trend, statistical analysis showed no significant difference across age groups. Similar findings have been reported by Maganira et al. (2018) in Ludewa district in the southern highlands of Tanzania and Acevedo-Nieto et al. (2017) in rural communities of eastern Minas Gerais, Brazil. These findings may be indicative of long exposure time to infection of adult pigs compared to growers. It is possible that adult pigs, potentially having more access to contaminated environments, are at a higher risk of infection. However, the lack of statistical significance could be attributed to small sample sizes in the different age groups.

Molecular analysis of cysticerci from various carcass organs in three infected pigs confirmed their identity as *T. solium* using agarose gel electrophoresis. This technique, known for its high sensitivity, allows differentiation of *T. solium* from other potential Taenia species infecting pigs. By amplifying unique genetic markers specific to *T. solium*, it provided conclusive evidence of its presence in pigs from Mpwapwa District, supporting similar studies in a nearby district (Maganira et al., 2019). Agarose gel electrophoresis is crucial, as *T. hydatigena* could cross-react with the genus-specific Ag-ELISA (Akoko et al., 2019; Deckers and Dorny, 2010). This molecular confirmation improves understanding of porcine cysticercosis transmission dynamics in the region, emphasizing the need for further research on other Taenia species in the disease's epidemiology. The findings highlight the importance of molecular diagnostic tools for accurate cysticercosis diagnosis, aiding in the development of effective control strategies and improving public and veterinary health outcomes.

Identified risk factors for porcine cysticercosis included pig management practices, household pig herd size, pig pen design, feed sources, ignorance of porcine cysticercosis and open-field defecation practices in the study. Analysis revealed significant associations between specific management practices and porcine cysticercosis seropositivity. Households keeping more than six pigs had 3.08 times higher odds of infection compared to households with fewer pigs, likely due to increased environmental contamination with *T. solium* eggs in larger herds. Similarly, semi-intensive pig farming systems were associated with higher odds of seropositivity compared to intensive systems, as limited confinement increases pigs' interaction with contaminated environments. Pigs housed in porous pens were 2.49 times more likely to be seropositive due to inadequate barriers preventing contact with contaminated areas. Ignorance of porcine cysticercosis and the presence of open-field defecation further exacerbated transmission risks, highlighting the role of poor sanitation in facilitating the spread of *T. solium* eggs. These findings are consistent with previous studies (Eshitera et al., 2012; Kajuna et al., 2022; Maganira et al., 2018; Maganira et al., 2019; Shonyela et al., 2017; Yohana et al., 2013). The presence of open-field defecation in the study area underscores poor toilet use, despite all surveyed households reportedly having either pit latrines or improved toilets. This suggests that owning a toilet does not guarantee its use by household members. Further investigation is needed to understand the reasons behind open-field defecation, which significantly increases the risk of pig infection. Additionally, the use of local feed and kitchen leftovers showed a strong association with seropositivity, consistent with findings from a study in Kiambu County, Kenya (Samuel et al., 2018). These results emphasize the need for careful feed preparation to reduce the risk of contamination. Ignorance about the disease perpetuates high-risk behaviors, necessitating a community-focused education campaign. Such efforts should integrate knowledge about the parasite's life cycle, zoonotic risks, and the economic burden of infections. Collaboration between veterinary and public health sectors can aid in designing culturally appropriate interventions, improving both pig management and sanitation practices to disrupt the parasite's transmission cycle effectively.

This study highlights key demographic factors likely influencing farming practices and porcine cysticercosis management in Mpwapwa District. The predominance of female respondents (82.4 %) reflects the significant role women play in agriculture, consistent with gender trends in rural Tanzania (Wilson and Swai, 2014). Most adult respondents (78.4 %) suggest a well-established farming community, though the smaller representation of younger individuals raises concerns about the future of farming and managing zoonotic diseases. Similar to findings by Sanga et al. (2023), 68.6 % of respondents in this study had formal education, which may support improved disease prevention practices, while the 31.4 % with informal education may face challenges in adopting new knowledge. Additionally, consistent with Sanga et al. (2023), the high percentage of respondents (90.2 %) with over three years of farming experience reflects a knowledgeable community, though the 9.8 % with less than two years of experience may need further training to address risks such as porcine cysticercosis.

### 4.1 Limitations of the study

Despite its contributions, this study has some limitations. The primary limitation is the use of Ag-ELISA, a widely employed diagnostic tool with an estimated overall sensitivity of 87 % and specificity of 95 %. However, its sensitivity decreases when detecting lower infection intensities of viable *T. solium* cysticerci. Additionally, in regions where other *Taenia* species, such as *T. hydatigena*, coexist, the assay's specificity may be compromised due to cross-reactivity with *non-T. solium* species. Future studies should incorporate more specific molecular diagnostic techniques to confirm infections. Additionally, this study did not assess parasite load, which could have provided insights into the severity of infections. The relatively small number of animals examined for cysts, slightly presents the genetic diversity of *T. solium* in the region. Future studies with larger sample sizes, longitudinal designs, and expanded molecular analyses are recommended. Furthermore, as this study was conducted during the dry season, future research should also consider the rainy season to account for potential seasonal variations in transmission dynamics. To strengthen the understanding of porcine cysticercosis transmission in Mpwapwa District, future research should investigate the presence of taeniasis in humans, particularly in households that keep infected pigs, as well as in the broader community. Integrating both human and animal epidemiological data will provide a comprehensive picture of *T. solium* transmission and inform targeted control strategies.

## 5 Conclusions

This study confirms the presence of porcine cysticercosis in Mpwapwa District and underscores the need for targeted interventions. Effective control strategies should focus on improving sanitation, adopting intensive farming systems, and enhancing biosecurity measures, such as constructing non-porous pig pens and restricting pigs' access to contaminated environments. Public health education for farmers on the transmission and prevention of porcine cysticercosis is crucial to reducing the disease burden. Additionally, further research with larger sample sizes is necessary to better understand risk factors and their interactions in endemic settings. Research is also needed to assess the level of poverty vis-a-vis piggery industry in Mpwapwa, as socio-economic factors may influence farming practices and the adoption of disease control measures.

## CRediT authorship contribution statement

**Justine Daudi Maganira:** Visualization, Software, Methodology, Formal analysis, Conceptualization, Writing – original draft, Validation, Resources, Investigation, Data curation. **Noel Mark Makwinya:** Project administration, Conceptualization, Writing – review & editing, Funding acquisition. **Beda John Mwang'onde:** Writing – review & editing, Resources, Conceptualization, Supervision, Methodology.

## Declaration of competing interest

The authors declare that they have no conflict of interest.

## Data Availability

The data used to support the results of this study are available from the corresponding author upon request.
